# Prevalence and predictors of metabolic syndrome among people living with human immunodeficiency virus (PLWHIV)

**DOI:** 10.1186/s13098-018-0312-y

**Published:** 2018-02-21

**Authors:** Dula Dessalegn Bosho, Lemessa Dube, Teshale Ayele Mega, Dawit Abera Adare, Mikyas Gashaw Tesfaye, Tesfahun Chanie Eshetie

**Affiliations:** 1 Department of Pharmacy, Faculty of Health Sciences, Institute of Health, Jimma University, Jimma, Ethiopia; 2 Department of Epidemiology, Public Health Faculty, Institute of Health, Jimma University, Jimma, Ethiopia; 3 Department of Medical Laboratory, Jimma University Medical Centre, Institute of Health, Jimma University, Jimma, Ethiopia

**Keywords:** Metabolic syndrome, HIV/AIDS, Ethiopia

## Abstract

**Background:**

Use of combination antiretroviral therapy (cART) has led to significant reductions in morbidity and mortality. However, there is a growing concern about metabolic syndromes (MS), among patients receiving cART. Despite this fact, there is limited evidence for the prevalence of the MS among HIV-infected persons receiving cART in developing countries, particularly Ethiopia.

**Objective:**

To determine the prevalence and predictors of MS among people living with HIV/AIDS in Jimma health centre, Jimma Zone south west Ethiopia.

**Methods:**

A cross-sectional study was conducted on people living with HIV/AIDS (PLWHA) in Jimma health centre that fulfilled the inclusion criteria. Data on demographic and anthropometric characteristics were collected using World health organization (WHO) stepwise approach. Fasting blood glucose and lipid profile was measured. The Third Report of National Cholesterol Education Program-adult treatment panel III (NCEP-ATP III)-2001, the International Diabetes Federation (IDF)-2005 and the Joint interim statement-2009 (JIS) criteria were used to define MS. Data were analyzed using statistical software package (SPSS) version 20.0. Logistic regression analysis was done to identify predictors of MS and predictors with p value < 0.05 were used to declare statistical significance.

**Results:**

Of 268 HIV-infected participants included in the analysis, 211 (78.7%) were women. The mean age of the participants was 39.32 ± 10.626 years. Using the NCEP-ATP III criteria, the prevalence of MS was found to be 23.5% (63 patients). While it was 20.5% (55 patients) and 27.6% (74 patients) with IDF and JIS criteria respectively. Enrollment in formal education resulted in 75% increment in the odds of MS (AOR = 0.25, 95% CI [0.072–0.879]). The odds of MS in patients with body mass index > 25 kg/m^2^ was elevated to 13.4 times (AOR = 13.39, 95% CI [3.943–45.525]) and exposure to D-drugs was attributed to 59% increment in the odds of MS (AOR = 1.59, 95% CI [0.58–4.56]), although the finding lacks statistical significance.

**Conclusions:**

Metabolic syndromes was relatively common to the study population. Hence, promoting health education and monitoring patient’s clinical and laboratory parameters at every visit and taking appropriate measure is ideal.

## Background

Metabolic syndromes (MS) are a cluster of biochemical and anthropometric abnormality that has high predictive ability for the development of atherosclerotic cardiovascular diseases (CVD) [1]. A variety of definitions had been used with different sets of diagnostic criteria, which vary from specific principal elements. But, it generally includes hypertension, obesity, glucose intolerance, hypertriglyceridemia and high density lipoprotein (HDL) dyslipidemia [2]. It is a constellation of cardiovascular risk factors of multifactorial etiology including the use of combination antiretroviral therapy (cART) [3].

Combination antiretroviral therapy (cART) has modified the natural history of human immunodeficiency virus (HIV) infection, leading to a significant reduction in morbidity and mortality. However, long-term toxicity and variety of metabolic abnormalities including dyslipidemia, fat redistribution, high blood pressure, and insulin resistance have frequently been reported, particularly when it contains protease inhibitors [4]. There is also a growing concern that, metabolic complications associated with HIV infection such as, hypertriglyceridemia, low HDL, and weight loss, continue to occur to untreated HIV-infected patients, while lipodystrophy, obesity, hypercholesterolemia, and insulin resistance are increasingly reported among those patients receiving cART [5].

Despite measurable achievements in reducing morbidity and mortality, the cART uses had also given a rise to metabolic and morphologic abnormalities which are risk factors of CVD. Recent finding indicated that, there is a high prevalence of MS among patients receiving ART [6]. However, other data suggested that, the increased prevalence of MS among HIV-infected patients may be more reflective of the burgeoning epidemic of obesity than a predominant effect of ART [7]. Results of the Data Collection on Adverse Events of Anti-HIV Drugs (DAD) study showed that, men were more likely than women to have MS and therapy with non-nucleotides reverse transcriptase inhibitors (NNRTIs) may be protective [8].

There is limited evidence for metabolic complications and risk factors of MS in HIV-infected patients in sub-Saharan region, particularly in Ethiopia [9]. Therefore, this study was aimed to investigate the prevalence and predictors of MS among people living with HIV/AIDS in Jimma Health Centre, Jimma Zone South-west Ethiopia.

## Method and participants

### Study design and study population

This was a cross-sectional study conducted at Jimma health center. The study population was sampled HIV patients attending the clinic for follow-up and on ART. The eligibility criteria for the study participants were; patients aged ≥ 18 years and on follow-up. We exclude patients that refused to sign informed consent or unwilling to participate and those who failed to fast for the next appointment. The health centre serves as an outpatient facility offering HIV care and treatment amongst other services. It is situated in Jimma town, 364 km away from the capital city, Addis Ababa and serves both rural and urban population. It provides services for around 800 HIV positive patients. The study was conducted between April 1 to May 30, 2016.

### Sample size calculation and selection of study participants

Sample size of 286 was estimated using a single population proportion formula with the following assumptions; 50% prevalence of metabolic syndrome among general population in Ethiopia, 5% margin of error, 95% confident interval and 10% for non-response. The study participants were selected using simple random sampling technique.

### Study variables

Metabolic syndromes among patients receiving cART was considered as an outcome variable and patient related (age, gender, educational level, income, occupation), diseases related (co-morbidities, CD4 count, WHO staging, duration HIV since diagnosed, opportunistic infections), behavioural related (drug use, lifestyle modification) and drug/therapy related factors (concomitant drug therapy, type of cART regimens, duration of cART) were considered as predictor variables.

### Data collection

An English version data collection tool adapted from WHO STEP wise approach to chronic disease risk factor surveillance [10, 11] and the patient charts were used to design the data collection tool. Data was collected on socio-demographic and clinical variables both from patients and their charts. The patients’ charts review was followed by collecting all necessary information about the patient. Tools used to collect data on variables such as, anthropometric (weight, height, waist circumference), blood pressure, and heart rate (HR) were standardized prior to be used for actual patient data collection. All measurements were conducted under the standard operating procedure by trained nurses with strict supervision of the principal investigator.

Blood pressure (BP) was measured as the average of the last two of three measurements with the Omron Automatic Inflation Blood Pressure Monitor taken at intervals longer than 2 min after the patient had been sitting for at least 30 min. Anthropometric measurements (waist circumference (WC) was measured with a flexible inelastic tape placed on the midpoint between the lower rib margin and the iliac crest in a perpendicular plane to the long axis of the body. Height was determined without shoes using a portable stadiometer. Weight was measured using a Tanita scale; patients were fully dressed, without heavy clothing or shoes). HR was recorded from the Omron Automatic Inflation Blood Pressure Monitor during blood pressure measurement. Similarly, biochemical measurements such as, fasting blood glucose (FBG), blood lipid, and creatinine were collected following laboratory standard operating procedures by a trained laboratory technologist. Blood sample was collected from each participant on the next morning after fasting for 12 h using vacutainer blood collection system to determine FBG and lipid profile. Five millilitre of blood was drawn from each patient’s cubital vein. Laboratory analysis of sample was done by laboratory technician. Serum samples were analyzed for FBG level and lipid profile [total cholesterol (TC), high density lipoprotein-cholesterol (HDL-c), low density lipoprotein-cholesterol (LDL-c) and triglycerides (TGs)] using ABX Pentra 400 machine. Enzymatic colorimetric assay method was used for the measurement of total cholesterol (CHODPAP method) and triglyceride (GPO-PAP method), while direct homogeneous enzymatic colorimetric assay technique was utilized for the measurement of HDL-c and LDL-c. FBG level was measured by glucose oxidase method (GOD-PAP).

### Data analysis and interpretation

Data were cleaned, coded and entered into Epi-Data version 3.1 and exported to the Statistical package for Social Science (SPSS) version 20.0 for analysis. Descriptive statistics using frequency distribution was performed for socio-demographic, epidemiological, clinical, and laboratory values. Association for the predictors and outcome variables were assessed using Chi square test and logistic regression analysis. Multivariate analysis using logistic regression was performed to control effect of confounding variables and to identify the independent predictors of MS. Predictor variables with p value of < 0.05 were used to declare statistically significance.

#### Outcome definition

The NCEP-ATP III 2001 guidelines define MS as having 3 or more of the following criteria: abdominal obesity (WC ≥ 102 cm for men and ≥ 88 cm for women); TG ≥ 1.7 mmol/L; HDL-c ≤ 1.03 mmol/L in men and ≤ 1.29 mmol/L in women; BP ≥ 130/85 mmHg or on treatment for hypertension; and FBG level of ≥ 6.1 mmol/L or on treatment for diabetes [12]. With IDF 2005-criteria, an individual is considered to have MS if WC ≥ 94 cm for men and ≥ 80 cm for women plus any of two or more of the following: TG ≥ 1.7 mmol/L; HDL–c ≤ 1.03 mmol/L in men and ≤ 1.29 mmol/L in women; BP ≥ 130/85 mmHg or on treatment for hypertension; and FBG level of ≥ 5.6 mmol/L or on treatment for diabetes [2]. Whereas the Joint Interim Statement (JIS) criteria, defines MS as having 3 or more of the following: WC ≥ 90 cm for men and ≥ 80 cm for women; TG ≥ 1.7 mmol/L or on treatment for TGs; HDL-c ≤ 1.0 mmol/L in men and ≤ 1.3 mmol/L in women or on treatment for HDL-c; BP ≥ 130/85 mmHg or on treatment for hypertension; and FBG level of ≥ 5.6 mmol/L or on treatment for elevated glucose will define MS [13].

## Results

Of 286 HIV-infected randomly selected patients, 18 of them declined to give informed consent and were excluded from the analysis.

### Socio-demographic characteristics of study participants

Among 268 HIV-infected participants included in the analysis, 211 (78.7%) were women. The mean age of participants was 39.32 ± 10.626 years. Majority of them (73.5%) were enrolled in formal education and, 64.2% of these were able to read and write. More than two-third of them (69%) had monthly income of ≤ 500 Ethiopian birr (ETB). About 15 (5.6%) of the participants had history of cigarette smoking and 4 (1.5%) were current smokers. Eighty-nine (33.2%) of them had history of Khat chewing and current chewers were about 17 (6.3%). Those with past history of alcohol consumption were about two-fifth (38.1%) and 38 (14.2%) of them currently drink alcohol 1–4 times per week. Nearly half of them (46.6%) could get fruits 1–2 days per week and majority of them (52.6%) were physically inactive (Table 1).

**Table 1:** Socio-demographic characteristic of people living with HIV/AIDS at follow up in Jimma health centre ART clinic, April 01 to May 30, 2016

Variables	Frequency, N = 268 (%)
Sex	
Male	57 (21.3)
Female	211 (78.7)
Age (years)	
18–30	60 (22.4)
31–40	109 (40.7)
41–50	61 (22.8)
> 50	38 (14.2)
Able to write and read	
No	96 (35.8)
Yes	172 (64.2)
Enrolled in formal education	
Yes	197 (73.5)
No	71 (26.5)
Monthly income in ETB	
≤ 500	185 (69.0)
501–1500	67 (25.0)
1501–2500	7 (2.6)
≥ 2501	9 (3.4)
Cigarette smoking history	
No	253 (94.4)
Yes	15 (5.6)
Current smoker	
No	264 (98.5)
Yes	4 (1.5)
Khat chewing history	
No	179 (66.8)
Yes	89 (33.2)
Current Khat chewer	
No	72 (26.9)
Yes	17 (6.3)
Current alcohol consumption	
No	166 (61.9)
Yes	102 (38.1)
Frequency of alcohol drink in past 12 months	
4 drinks per week	38 (14.2)
2 drinks per week	38 (14.2)
Less than one a month	26 (9.7)
Fruit intake	
None	78 (29.1)
1–2 days/week	125 (46.6)
3–4 days/week	41 (15.3)
5–7 days/week	24 (9.0)
Physical activity	
Inactive	141 (52.6)
Active	127 (47.4)

*ETB* Ethiopian birr

### Clinical and drug related characteristics

One hundred and seventeen (43.7%) patients had reported that their BP was measured. About 36.6% of them had their BP measured in the past 12 months. Around 17 (6.3%) of them reported that they had been told by health care professional they had hypertension. Of these, 5 (1.9%) were receiving anti-hypertensive medication in the past 2 weeks. About 3.4% of patients were asthmatic and 3 (1.1%) had chronic kidney diseases.

Nearly half of the patients (48%) were WHO stage 2 and majority of them (53.7%) had lived 6 years and above with HIV/AIDS. Most of them (83.2%) had CD4 count less than 350 cell/mm^3^ at baseline and 226 (84.3%) had opportunistic infections (OIs) prior to HIV diagnosis. More than half (52.6%) of them were receiving TDF + 3TC + EVF at base line and about 60.8% of them were taking ART for less than 5 years. Moreover, 52.6% of the participants were receiving cotrimoxazole for prevention of OIs together with cART (Table 2).

**Table 2:** Clinical and drug related characteristics of people living with HIV/AIDS at follow up in Jimma health centre ART clinic, April 01 to May 30, 2016

Variables	Frequency N = 268 (%)
History of BP measured by health workers	
No	151 (56.3)
Yes	117 (43.7)
Period of blood pressure measured	
The past 12 months	98 (36.6)
Within past 1–5 years	16 (6.0)
More than past 5 years	3 (1.1)
Previous history of hypertension	
No	251 (93.7)
Yes	17 (6.3)
Taking antihypertensive agent past 2 weeks	
No	16 (4.5)
Yes	5 (1.9)
Previous history of asthma	
No	259 (96.6)
Yes	9 (3.4)
Previous history of chronic kidney diseases	
No	265 (98.9)
Yes	3 (1.1)
Duration of HIV diagnosis	
≤ 5 years	122 (45.5)
≥ 6 years	146 (54.5)
CD4 (cell/mm^3^)	
< 350	223 (83.2)
351–550	38 (14.2)
≥ 551	7 (2.6)
WHO stage	
I	48 (17.9)
II	129 (48.1)
III	83 (31)
IV	8 (3)
OIs prior to HIV diagnosis	
Yes	226 (84.3)
No	42 (15.7)
Types of cART regimes at baselines	
D-drugs^a^	92 (34.3)
Others^b^	176 (65.7)
Duration of cART (years)	
≤ 5	163 (60.8)
≥ 6	99 (36.9)
Others^c^	6 (2.2)
Concomitants drug use	
Cotrimoxazole	141 (52.6)
INH	30 (11.2)
Multivitamins	6 (2.2)
Others^d^	13 (4.9)

*BP* blood pressure, *HIV* human immunodeficiency virus, *CD4* cluster differentiation 4, *OIs* opportunistic infections, *URTIs* upper respiratory tract infections, *cART* combination antiretroviral therapy, *d4T* stavudine, *3TC* lamivudine, *AZT* zidovudine, *TDF* tenofovir, *NVP* nevirapine, *EVF* efavirenz, *INH* isoniazid

^a^d4T + 3TC + NVP; d4T + 3TC + EVF

^b^AZT + 3TC + NVP; AZT + 3TC + EVF; TDF + 3TC + EVF; TDF + 3TC + NVP

^c^Naïve patients

^d^Enalapril, hydrochlorothiazide, atenolol, prednisolone, salbutamol puff

### Physical, biochemical measurements and prevalence of MS

Physical and biochemical measurements indicated that, nearly two-fifth (38.4%) of the patients had hypertension and almost more than two-third of them (68.5%) had normal body weight. About 18.7% of the patients’ WC was in line with the definition of MS. HDL-c was the most prevalent components of MS 132 (49.3%) followed by BP 103 (38.4%). Therefore, according to NCEP-ATP III, the prevalence of MS was 63 (23.5%), and it was 55 (20.5%) according to IDF. Similarly, the prevalence of MS was 74 (27.6%) according to JIS (Table 3).

**Table 3:** Physical and biochemical measurements among people living with HIV/AIDS at follow up in Jimma health centre ART clinic, April 01 to May 30, 2016

Variables	Frequency, N = 268 (%)	Components of MS
Blood pressure		
SBP ≥ 130 mmHg and DBP ≥ 85 mmHg	103 (38.4%)	103 (38.4%)
BMI (kg/m^2^)		
< 18.5	50 (18.7)	
18.5–24.9	184 (68.7)	
≥ 25	34 (12.7)	
WC		
Male ≥ 102 cm	2 (0.7)	
Female ≥ 88 cm	48 (17.9)	50 (18.7)
FBG ≥ 100 mg/dl	46 (17.2)	46 (17.2)
TC ≥ 200 mg/dl	67 (25.0)	
HDL-c ≤ 40 mg/dl male and ≤ 50 mg/dl female	132 (49.3)	132 (49.3)
TGs ≥ 150 mg/dl	80 (29.9)	80 (29.9)
LDL-c ≥ 100 mg/dl	101 (37.7)	

*SBP* systolic blood pressure, *DBP* diastolic blood pressure, *BMI* body mass index, *WC* waist circumference, *FBG* fasting blood glucose, *TC* total cholesterol, *HDL* high density lipoprotein-cholesterol, *TGs* triglycerides, *LDL* low density lipoprotein-cholesterol

### Predictors of metabolic syndrome

The result of binary logistic regression analysis of the independent predictors showed that age group 41–50 years and those not enrolled in formal education were significantly associated with MS (COR = 3.171, 95% CI 1.268–7.928, and COR = 0.384, 95% CI 0.179–0.827) respectively. In addition, monthly income level above 2501 ETB (COR = 4.141, 95% CI 1.007–17.02) was also associated with MS. Moreover, physical activity (COR = 0.558, 95% CI 0.312–0.999), duration of HIV diagnosis less than 5 years (COR = 0.286, 95% CI 0.151–0.543), body mass index ≥ 25 kg/m^2^ (COR = 10.47, 95% CI 3.513–31.24), duration of ART regimens for more than 6 years (COR = 3.011, 95% CI 1.676–5.410), and type of cART regimens (COR = 2.927, 95% CI 1.635–5.238) were significantly associated with MS.

Multivariable logistic regression analysis was done for all explanatory variables with p < 0.25 in bivariate logistic regression analysis. Accordingly, those not enrolled in formal education and body mass index above 25 kg/m^2^ were independent predictors of MS. Therefore, those who were not enrolled in formal education were less likely to have MS (AOR = 0.252, 95% CI 0.072–0.879) and overweight patients were 13.4 times more likely to have MS (AOR = 13.398, 95% CI 3.943–45.525) (Table 4).

**Table 4:** Predictors of MS among PLWHIV at follow up in Jimma health centre ART clinic, April 01 to May 30, 2016

Variables	MS according to (NCEP-ATP III)		Bivariate analysis		Multivariate analysis	
			p value	COR (95% CI)	p value	AOR (95% CI)
	Yes	No				
Sex						
Male	12	45	0.623	0.837 (0.411–1.703)		
Female	51	160	1	1		
Age in years						
18–30	8	52	1	1	1	1
31–40	24	85	0.172	1.835 (0.768–4.387)	0.538	1.358 (0.513–3.594)
41–50	20	41	0.014	3.171 (1.268–7.928)	0.175	2.058 (0.725–5.847)
≥ 51	11	27	0.062	2.648 (0.953–7.362)	0.344	1.782 (0.539–5.894)
Able to write or read						
No	17	79	0.097	0.589 (0.316–1.099)	0.979	1.014 (0.355–2.899)
Yes	46	126	1	1	1	1
Formal education						
Yes	54	143	1	1	1	1
No	9	62	0.014	0.384 (0.179–0.827)	0.031	0.252 (0.072–0.879)
Smoking history						
No	61	192	1	1		
Yes	2	13	0.349	0.484 (0.106–2.206)		
Past Khat chewer						
No	46	133	1	1	1	1
Yes	17	72	0.232	0.683 (0.365–1.277)	0.583	0.813 (0.388–1.704)
Current Khat chewer						
No	14	58	1	1		
Yes	3	14	0.865	0.888 (0.224–3.517)		
Alcohol consumption						
No	39	127	1	1		
Yes	24	78	0.995	1.002 (0.560–1.792)		
Frequency of alcohol						
1–4 days/weeks	11	27	1	1		
1–3 days/weeks	8	30	0.428	0.655 (0.229–1.868)		
< Once a month	5	21	0.381	0.584 (0.176–1.943)		
Physical activity						
Inactive	40	101	1	1	1	1
Active	23	104	0.050	0.558 (0.312–0.999)	0.325	0.702 (0.347–1.420)
Fruit intake						
None	23	55	1	1	1	1
1–2 days/week	27	98	0.206	0.659 (0.345–1.258)	0.103	0.513 (0.230–1.145)
3–4 days/week	9	32	0.380	0.673 (0.277–1.630)	0.621	0.767 (0.269–2.190)
5–7 days/week	4	20	0.220	0.478 (0.147–1.554)	0.334	0.513 (0.132–1.986)
Comorbid disease						
No	53	187	1	1	1	1
Yes	10	18	0.112	1.960 (0.854–4.500)	0.543	1.383 (0.486–3.930)
Duration of HIV						
≤ 5 years	15	107	< 0.001	0.286 (0.151–0.543)	0.136	0.482 (0.185–1.259)
> 5 years	48	98	1	1	1	1
CD4 (cell/mm^3^)						
< 350	53	170	1	1		
351–550	9	29	0.991	0.995 (0.443–2.235)		
≥ 551	1	6	0.566	0.535 (0.063–4.541)		
WHO stages						
Stage I	12	35	1	1		
Stage II	28	101	0.592	0.809 (0.371–1.760)		
Stage III	21	63	0.946	0.972 (0.428–2.209)		
Stage IV	2	6	0.975	0.972 (0.172–5.481)		
Duration of cART						
≤ 5 years	26	137	1	1	1	1
> 5 years	36	63	< 0.001	3.011 (1.676–5.410)	0.372	1.563 (0.586–4.173)
Others^a^	1	5	0.963	1.054 (0.118–9.394)	0.735	1.499 (0.143–15.666)
Types of cART						
D-drugs^b^	33	56	< 0.001	2.927 (1.635–5.238)	0.366	1.591 (0.581–4.360)
Others^c^	30	149	1	1	1	1
BMI (kg/m^2^)						
< 18.5	6	44	1	1	1	1
18.5–24.9	37	147	0.195	1.846 (0.731–4.660)	0.129	2.191 (0.795–6.036)
≥ 25	20	14	< 0.001	10.47 (3.513–31.24)	< 0.001	13.398 (3.943–45.525)

*COR* crude odd ratio, *AOR* adjusted odd ratio, *HIV* human immunodeficiency virus, *CD4* cluster determinant 4, *WHO* world health organization, *cART* combination antiretroviral therapy, *BMI* body mass index, *d4T* stavudine, *3TC* lamivudine, *AZT* zidovudine, *TDF* tenofovir, *NVP* nevirapine, *EVF* efavirenz

^a^Naïve patients

^b^d4T + 3TC + NVP/or EVF

^c^AZT/or TDF + 3TC + NVP/or EVF and naïve patients

## Discussion

In this study, the prevalence of MS was 23.5, 20.5 and 27.6% by NCEP-ATP III, IDF and JIS respectively. The JIS result of MS in our finding was higher than the others two. This finding was consistent with the study conducted in Malaysia (26.5, 37.4 and 43.4%) [13] and South Africa (24.1, 25.5, and 28.2%) respectively [14]. The prevalence of MS according to JIS in our study was in closer to the finding from South Africa i.e. 28.2% [14]. NCEP-ATP III did not use any single risk factor (e.g. abdominal obesity) as a requirement for MS diagnosis. The IDF clinical definition thus uses the presence of abdominal obesity necessary for diagnosis. If this is the case, 2 additional factors originally listed in the NCEP-ATP III definition are sufficient to diagnose MS [15]. This makes variation in NCEP-ATP III and IDF definition of MS.

Based on NCEP-ATP III criteria, our finding was higher than results obtained from Darlinghurst and Southern Brazil, in which the prevalence of MS was 18, 17.2% respectively [16, 17]. Our finding was also higher than the study from Hawassa University Referral Hospital and Jimma University specialized hospital, where the prevalence of MS was 18.1 and 21.1% respectively [18, 19]. This variation might be due to the fact that, there were higher numbers of participants with low HDL and high blood pressure in our study. The other possible explanation might be our study was done in primary health care setting unlike that of Hawassa and JUSH, which are referral settings with high level of care and professional profile. On the other hand, our finding was in line with the study from Thailand in which 22.2% of participants had MS [5]. Our finding was lower than the results obtained from Danish, where 27% of the participants were found to be victims of MS [20]. This difference might be due to the variation in different components of MS (WC, high FBG and TGs) which was lower in our study and the small sample size utilized in our study.

The prevalence of MS using IDF criteria in our study was higher than the study conducted in Darlinghurst (i.e. 14%) [16]. This variation might be due to the higher proportions of women (78.7%) in our study and WC can be considered as basic parameters for MS diagnosis in IDF criteria. However, the Darlinghurst study considered only men patients. In contrary, the prevalence of MS using IDF criteria in our finding was lower than the finding from Hawassa university referral hospital [18]. This discrepancy might be due to use of abdominal obesity as basic requirement for diagnosing MS by IDF criteria. There were lower proportions of patients that had high abdominal obesity in our finding.

This study revealed that, being enrolled in formal education was independent predictor of MS. Similar finding was also reported from Nairobi [21]. This could be justified by the higher proportions (72.8%) of patients’ enrolled in formal education were receiving D-drugs, which were well known risk of MS as reported by other study [18]. The prevalence of MS increased with age [22], but age groups were not predictors of MS in our finding, unlike reports from Hawassa University referral hospital and Nairobi [18, 21]. This difference might be due to variation in age group (i.e. more than three-fifth of them had < 40 years old in our study).

The participant’s characteristics like smoking habit, alcohol consumption, physical activity and Khat chewing were not identified as independent predictors’ of MS. This was consistent with finding from Hawassa University referral hospital [18]. This study also showed that, body mass index of ≥ 25 kg/m^2^ was independent predictor of MS. This was in line with the study done in Hawassa University referral hospital [18].

The most frequently observed features of abnormalities were lower HDL (49.3%), raised BP (38.4%) and raised LDL (37.7%), while TGs (29.9%), TC (18.7%) and FBG (17.2%) were less common.

The development of cART-associated MS is complex and a number of factors including direct effects of ART on lipid metabolism, endothelial and adipocyte cell function, and mitochondria [23] could be implicated. Cumulative evidence has pointed out the relationship between different metabolic disorders and cART use, including insulin resistance, hyperlipidemia, and lipodystrophy, even though it remains controversial whether this effect can be directly ascribed to cART [24]. Although capturing the impact on duration of specific drug on the development of MS could be impossible, it seemed cART duration could have an impact. This might be hypothesized by our finding that, patients who received cART more than 5 years has 15.6% higher odds of having MS than those below 5 year, although our finding lacked statistical significance. Similar results were reported by study done in Hawassa University referral hospital [18]. In contrast, study done in Jimma University specialized hospital had witnessed the impact of long term exposure (> 12 months) of cART on MS [19]. This difference might be due to variation in years of cART duration categories in the statistical analysis. Likewise, the odds of having MS was increased by 15.9% among patients’ treated with D-drugs, however, the difference was statistically insignificant. The study conducted in Hawassa University referral hospital, showed that receiving D4T − 3TC − EFV regimen was significantly associated with higher odds of MS [18].

As one of its strength, this study tried to high light the prevalence and some of the risk factors of MS in low income settings, which was not fully understood in HIV-infected patients. However, the anticipation of early diagnosis for its components should be part of the good clinical practices. Consequently, doing so might contribute to slow or even reduce its impact on the development of CVD. However, our study had some limitation. Being a single center study and focused on primary care setting could affect the generalizability.

## Conclusions

Metabolic syndromes were relatively common to HIV/AIDS patients in a primary care setting. In our finding one in four of the study participants had MS. Low level of HDL-c and high BP as a component of the five MS criteria were more prevalent in our study population. The study revealed that enrollment in formal education and overweight were independent predictors of MS.
